# Soiled airway tracheal intubation and the effectiveness of decontamination by paramedics: a randomised controlled manikin study protocol

**DOI:** 10.29045/14784726.2018.12.3.3.16

**Published:** 2018-12-01

**Authors:** Richard Pilbery, M. Dawn Teare, Mark Millins

**Affiliations:** Yorkshire Ambulance Service NHS Trust: Orcid ID: 0000-0002-5797-9788; University of Sheffield; Yorkshire Ambulance Service NHS Trust

**Keywords:** airway management, paramedic, suction assisted laryngoscopy and decontamination (SALAD), tracheal intubation

## Abstract

Vomiting and regurgitation are commonly encountered in out-of-hospital cardiac arrest with a reported incidence of 20–30%. Arguably, tracheal intubation is the preferred airway management technique in patients with ongoing airway contamination, but there is evidence that this is difficult to achieve when the airway is soiled. In addition, traditional suctioning techniques have been criticised, and training in the management of contaminated airways is limited. If standard suctioning techniques are not sufficient to maintain a clear airway and provide ventilation, then these patients will die, irrespective of the quality of chest compressions and the timeliness of defibrillation. This has led to the development of a combined suction/laryngoscopy technique to facilitate intubation, known as Suction Assisted Laryngoscopy and Airway Decontamination, and the creation of modified airway manikins to allow for practice in these techniques. However, to date there has only been one study specifically looking at the Suction Assisted Laryngoscopy and Airway Decontamination technique, and the outcomes were self-reported confidence measures of trainees in using the technique.

The primary objective of Soiled Airway Tracheal Intubation and the Effectiveness of Decontamination is to determine the difference between paramedic first-pass intubation success, before and after Suction Assisted Laryngoscopy and Airway Decontamination training, in a simulated soiled airway. The primary outcome is the difference in proportions of paramedic first-pass intubation success, before and after Suction Assisted Laryngoscopy and Airway Decontamination training.

Paramedic recruitment commenced in July 2018 and the study will enrol 154 paramedics by the end of 2018. The results of this study will contribute to the evidence relating to the Suction Assisted Laryngoscopy and Airway Decontamination technique.

## Introduction

Yorkshire Ambulance Service NHS Trust (YAS) has recently taken part in AIRWAYS-2, a randomised controlled trial, comparing the i-gel supraglottic airway device (SAD) versus tracheal intubation in the initial management of the airway in out-of-hospital cardiac arrest (OHCA) (Benger et al., 2018). This has provided an opportunity for closer scrutiny of advanced airway management by paramedics within the Trust, and has highlighted the challenge of intubation in the face of serious and/or ongoing airway contamination by blood or vomit. Anecdotally, it appears that traditional suctioning and airway clearing techniques may be ineffective in this group of patients.

Vomiting and regurgitation are commonly encountered in out-of-hospital cardiac arrest (OHCA) with a reported incidence of 20–30% (Simons, Rea, Becker, & Eisenberg, 2007; Voss et al., 2014). This is of concern since patients who have suffered an OHCA are already in extremis. If standard suctioning techniques are not sufficient to maintain a clear airway and provide ventilation, then these patients will die, irrespective of the quality of chest compressions and the timeliness of defibrillation. Arguably, tracheal intubation is the preferred airway management technique in patients with ongoing airway contamination, but there is evidence that this is difficult to achieve when the airway is soiled (Sakles et al., 2017). Even if patients survive to the hospital, it is possible that aspiration pneumonias may adversely affect survival, although this has yet to be proved empirically (Christ et al., 2016).

Traditional suctioning techniques have been criticised, and training in the management of contaminated airways is limited. This has led to the development of a combined suction/laryngoscopy technique to facilitate intubation, known as Suction Assisted Laryngoscopy and Airway Decontamination (SALAD), and the creation of modified airway manikins to allow for practice in these techniques (DuCanto, Serrano, & Thompson, 2017).

However, to date there has only been one study specifically looking at the SALAD technique, and the outcomes were self-reported confidence measures of trainees in using the technique. Other techniques have been described to manage significant airway contamination, including the use of a meconium aspirator (Kei & Mebust, 2017), which is not practical in the out-of-hospital environment (and requires a device that is not typically carried by UK ambulance services), and deliberate intubation of the oesophagus (the oesophageal diversion manoeuvre), of which the sum total of evidence in support of the procedure is a single case report (Kornhall, Almqvist, Dolven, & Ytrebø, 2015).

### Aims, objectives and the research question

This study aims to determine whether a short teaching session of the SALAD technique to paramedics improves their ability to intubate a contaminated airway.

The research question is: Does paramedic first-pass intubation success of a simulated contaminated airway improve following training in SALAD?

The primary objective is to determine the difference between paramedic first-pass intubation success, before and after SALAD training, in a simulated soiled airway. The primary outcome is the difference in proportions of paramedic first-pass intubation success, before and after SALAD training.

The secondary objective is to determine the difference in time taken to achieve first-pass intubation success, before and after SALAD training in a simulated soiled airway, and the effect of multiple intubation attempts on success rates following SALAD training. The secondary outcomes are:

- the mean of the differences in intubation attempt times, between first and second intubation attempts, and between pre- and post-training attempts; and
- the difference in success rates between participants who have two post-training intubation attempts versus participants who only have one post-training intubation attempt.

Arguably, overall intubation success could also be used as an indication of intubation competence, but by not allowing multiple intubation attempts at each stage of the assessment, the participants are minimally inconvenienced in terms of time required to take part in the study, while still providing an objective measurement of the effect of the training intervention. In addition, there are published values on non-physician first-pass intubation success, which is reported to be approximately 70%, although this is likely to be lower in soiled airways (Crewdson, Lockey, Røislien, Lossius, & Rehn, 2017).

## Methods

### Trial management

This study has been approved by the Health Research Authority and is registered with ClinicalTrials.gov (https://clinicaltrials.gov/ct2/show/NCT03599687). YAS is the sponsor of this study as the employer of the Chief Investigator (CI) and will undertake all sponsor responsibilities outlined in the UK Policy Framework for Health and Social Care Research.

There will be no trial management committee for this study.

### Trial design

The trial has been designed as a randomised controlled trial (RCT), with the primary objective of determining whether first-past intubation success rates are higher in the post-SALAD training group of participants. In order to adjust for changes in participant performance by making repeated attempts at intubation, paramedics will be randomised into either: making two pre-training intubation attempts and one post-training attempt (AAB); or making one pre-training intubation attempt and two post-training attempts (ABB).

This study will be conducted at a single site (YAS). All sessions will be conducted on Trust premises, typically ambulance stations or other training facilities around the Trust, which are geographically convenient for participants to attend. Participants will be NHS staff who are employed by YAS, and who are Health and Care Professions Council (HCPC) registered paramedics. In addition, they must be authorised to intubate within the Trust and must not have received any SALAD training in the last 3 months. Participants will be ineligible if they do not meet the inclusion criteria, are allergic to the ‘vomit’ ingredients or are unwilling to provide consent to participate.

### Recruitment

Potential participants will be invited indirectly to participate via Trust email and the weekly operational update that is widely distributed throughout the Trust. This will include simultaneous distribution of the participant information sheet (PIS) via the same route.

Paramedics who are interested in the study will be asked to contact the CI to arrange attendance at a study training session. The CI will confirm that the potential participant is an operational paramedic working for the Trust, and is not allergic to the ‘vomit’ ingredients. This will be documented on a spreadsheet of potential participants.

### Consent

All participants will have capacity as they are operational paramedics and employees of YAS. At the start of the study training session, informed consent will be obtained by the CI and verified by completion of a signed consent form.

### Withdrawal criteria

Participants can withdraw from the trial at any time and do not have to provide justification for doing so, by contacting the CI for the trial. Details of the withdrawal will be entered onto the case report form (CRF). Participants who withdraw can request that any non-anonymised data are erased.

Since the sample size is required to ensure the study is adequately powered, additional paramedic participants will be recruited, if necessary, to offset any withdrawals. For the same reason, this study will continue until the sample size has been reached.

### Intervention under study

#### SALAD manikin

The manikin to be used in the study is a modified TruCorp AirSim Advance, which has realistic airway anatomy and can be used for tracheal intubation training. The oesophagus of this manikin has been connected, via a hosepipe, to a bilge pump that is sited within a reservoir of simulated vomit. The vomit is water, coloured with food-grade colouring, and thickened with xanthan gum (a food additive). Once the bilge pump is switched on, it can generate a constant flow of liquid into the oropharynx, obscuring any view of the laryngeal inlet. The flow rate is controlled by a tap, which will be calibrated to provide 1 L/min of vomit to the oropharynx of the manikin during the intubation attempts. To keep vomit within the oropharynx, the left and right bronchi on the manikin have been occluded.

An endoscope camera will be placed in the posterior oropharynx after each intubation attempt and connected to a laptop, to provide a view of the vocal cords for the researcher, but not the participant, allowing confirmation of correct tube placement, while not dislodging the tube.

Standard intubation equipment, including personal protective equipment (PPE) and motorised suction, that is routinely used within YAS will be provided for participants, and the study researcher will act as a competent assistant for the intubation attempts.

#### Procedure

Informed consent will be obtained from paramedic participants prior to commencing the study. They will then be randomised to determine the order in which they will attempt intubation, and whether they will make two pre-training and one post-training attempts (AAB group), or one pre-training and two post-training attempts (ABB group). All attempts will utilise direct laryngoscopy, which is the standard intubation technique within YAS. Prior to each intubation attempt, the manikin will be primed with vomit. Once the oropharynx is full, the participant will undertake their first intubation attempt. The manikin will deliver vomit to the oropharynx at a rate of 1 L/min.

All intubation attempts will be video recorded to allow for accurate time-keeping, since the researcher will be assisting the paramedic with their intubation attempt. However, the researcher will also time the intubation attempt using a stopwatch to record the time, in the event that a video recording fails. Participants will be deemed to have begun their intubation attempt once the pump which makes the manikin vomit is turned on. The attempt will be considered over when:

- the paramedic who has intubated the manikin verbally confirms with the researcher that the attempt has been completed; or
- 90 seconds have elapsed; or
- the tracheal tube is placed into the oesophagus and the cuff is inflated while the pump is still running.

If the tracheal tube is not in the trachea, with the cuff inflated and connected to a bag-valve device within 90 seconds, the attempt will be considered a failure.

Participants randomised to the two pre-training attempts (AAB) will make a second intubation attempt prior to the group training session. Once all participants have completed their pre-training intubation attempt, the training session will be delivered, and will take around 45 minutes to complete, including time for participant practice. The training intervention will adopt the Advanced Life Support Group/Resuscitation Council 4-stage approach of skills teaching, and is comprised of (Bullock, Davis, Lockey, & Mackway-Jones, 2008):

1. a real-time demonstration of the SALAD technique by the researcher;
2. a repeated demonstration with an explanation of the rationale of the steps taken when performing SALAD (not real-time);
3. another demonstration of the SALAD technique conducted by the researcher, but guided by one of the participants; and
4. an attempt by the same participant who guided the researcher in the previous step, followed by a practice attempt by the other participants.

Following the training session, participants will make their post-training intubation attempt(s). This will be conducted using the same method as for the pre-training intubation attempt(s). Participants randomised into the two post-training attempts (ABB) will make their second attempt immediately following the first post-training attempt.

### Statistics and data analysis

#### Sample size calculation

The null hypothesis (H_0_) for this study is that the training intervention will have no effect on participant intubation success. The alternative hypothesis (H_1_) is that intubation success will change following the training intervention.

A sample size of 154 participants is required to determine a change in the proportion of intubation success, from 0.25 in the pre-training group, to 0.50 in the post-training group, with a power (1-β) of 90% and a significance level (β) of 5%. Given that there is no literature to guide expected performance, a conservative estimate has been made in consultation with an internationally recognised SALAD expert, Dr James DuCanto (J. DuCanto, personal communication, 26 April 2018).

**Figure 1.**
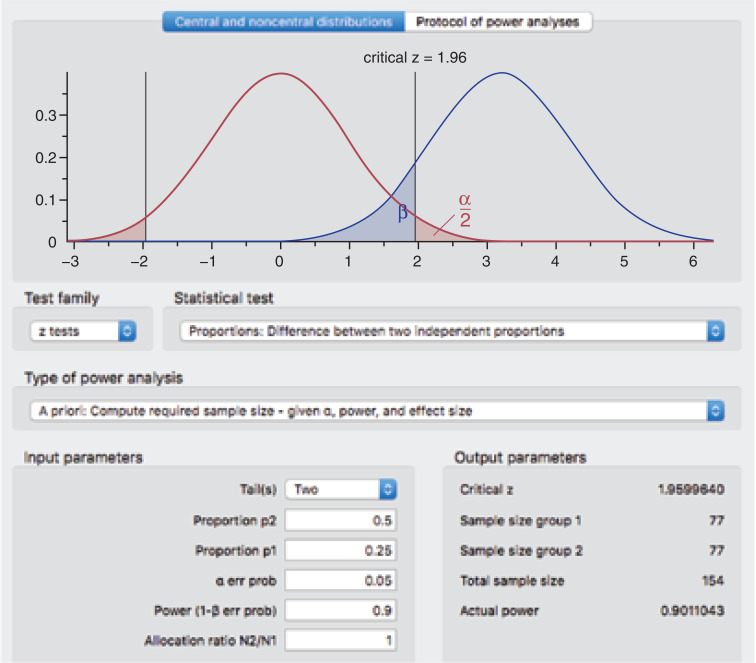
G*Power parameters for study sample size calculation.

The sample size calculation was determined by using the application G*Power, version 3.1, using the parameters and test shown in Figure 1. No subgroup analyses or adjustment of the analysis to account for the demographic data obtained from participants will be undertaken.

#### Statistical analysis plan

Descriptive statistics will be used to summarise the demographic data provided by participants. The order pathway that participants make their intubation attempts (AAB or ABB) will be 1:1 randomised, using a block randomisation sequence provided by RANDOM.ORG. To distinguish between the training pathways and number of the assessed attempts, we will use A_01_A_02_B_01_ and A_11_B_11_B_12_ to differentiate between groups and attempts.

##### Primary outcome analysis

To determine if the training has an effect and increases the success rate of intubation, we will compare the proportions of success in the groups who receive no training before their second intubation attempt (A_02_) with those who do receive training before their second intubation attempt (B_11_). Comparing the rates at these time points controls for any learning effect due to participants making more than one attempt at intubation. The difference in the two proportions will be analysed using a two independent samples proportion z-test. We will assume a two-sided type 1 error rate of 5%, and report the proportions and the difference in the proportions along with 95% confidence intervals.

##### Secondary outcome analysis

Intubation times will be truncated at 90 seconds. We will compare the mean of the differences (A_01_–A_02_) with the mean of the differences (A_11_–B_11_). We will also compare the mean of the differences seen at the final measurements, (A_01_–B_01_) and (A_11_–B_12_), to see if there are differences between the two pathways, which might suggest that practice following the training further improves the time to successful intubation. In addition, we will also compare the success rates between B_01_ and B_02_ to see whether practice following training improves intubation success rate. A student’s t-test will be utilised to test for the differences between mean pre- and post-training intubation attempt times. The difference in success rates will be analysed using a two independent samples proportion z-test. We will assume a two-sided type 1 error rate of 5%, and report the proportions and the difference in the proportions along with 95% confidence intervals.

#### Procedure(s) to account for missing or spurious data

In the event that the video recording cannot be used to determine intubation success and time to success, the researcher recorded outcomes will be utilised instead. Should a participant not complete all three attempts, then their data will not be included in the final analysis and a replacement participant sought to ensure that the target sample size is achieved.

### Data management

The source data for the study will consist of a custom CRF and video recordings. The CRF will be completed by the CI during the participant attempts. This will include the basic demographic data, whether the first-pass intubation is successful, if the tube was suctioned prior to an attempt at ventilation and the time taken to intubate.

To maintain participant confidentiality, the CRF will not contain any personal-identifiable data. Instead, the participant will be identified by a unique study ID. Video recordings of the training sessions will be held securely on a Trust computer which only the researcher can access. Once timings and confirmation of intubation success have been determined following review of the video, the recording will be erased (within one week, typically). Participants will not be identifiable from the data produced by the trial. All CRFs will be securely stored on Trust premises in a locked cabinet, within a secure room.

Direct access will be granted to authorised representatives from the sponsor, host institution and the regulatory authorities to permit trial-related monitoring, audits and inspections in line with participant consent.

Archiving will be authorised by the sponsor following submission of the end of trial report. All source documentation will be archived on Trust premises in a locked cabinet in a secure location. Video recordings will not be archived as they will be securely erased during the study.

Data will be available for future analysis for a period of 2 years prior to being archived. All essential documents will be archived for a minimum of 5 years after completion of trial. Destruction of essential documents will require authorisation from the sponsor. The study may be audited as part of the routine audit process as laid out in the Sponsors Research Governance Policy. All source documents and essential documents will be available to the sponsor for audit for at least 5 years.

### Ethical and regulatory considerations

Prior to the start of the trial, approval will be sought from a Research Ethics Committee (REC) for the trial protocol, informed consent forms and other relevant documents. Substantial amendments that require review by REC will not be implemented until the REC grants a favourable opinion for the trial, and all correspondence with the REC will be retained in the Trial Master File. If the trial is ended prematurely, the CI will notify the REC, including the reasons for the premature termination.

#### Data protection and participant confidentiality

All investigators and trial site staff will comply with the requirements of the General Data Protection Regulations 2018 with regards to the collection, storage, processing and disclosure of personal information.

Each participant will be allocated a participant ID once informed consent has been obtained. This will be used on all study source documents. Access to the source documents will be restricted to YAS Research and Development personnel only. Video recordings will only be accessible by the CI, the data custodian.

The CI and the statistician will be the only personnel to have access to the full dataset for the purposes of this study analysis. However, after the trial, an anonymised version of the dataset will be made available for other researchers.

### Peer review

Peer review has been provided as part of the funding application process and was proportionate, independent and expert.

### Public and patient involvement

YAS is fortunate to have a Patient Research Ambassador (PRA) who has been involved in the production of lay summaries and the sense-checking of documents, in addition to advising on the proposed dissemination plan.

### Protocol compliance

Any breach of protocol will be reported via the YAS Datix system to notify the Research and Development department at YAS. Duty of Candour will be considered in consultation with the YAS Duty of Candour specialist.

## Dissemination

The results of the study will be published in a peer-reviewed journal (*British Paramedic Journal*) and presented at relevant conferences such as the College of Paramedics national conference, 999 EMS Research forum and EMS2019. In addition, plain English summaries will be published in the College of Paramedics newsletter and free ambulance magazines, which are routinely posted to all ambulance stations in the UK. A summary report will be presented to the participants and YAS, and will be available on the study website (https://satiated.ambulanceresearch.co.uk). Finally, the ubiquity of social media will be utilised to highlight the publication of the study on Twitter, Facebook and other social media outlets.

Since part of the dissemination strategy involves publishing in peer-reviewed journals, author eligibility will be determined in accordance with the International Committee of Medical Journal Editors (ICMJE) authorship criteria. Professional writers will not be used.

## Conclusion

The SALAD technique has been widely promoted on social media as part of the Free Open Access to Medical Education (FOAMEd) movement. However, it is important that there is research to support methods such as SALAD and this study will assist in building the evidence base for SALAD.

Prior to undertaking clinical studies, it is important to determine the feasibility of teaching this technique to paramedics in a brief training session, and testing whether it has a beneficial effect on paramedic intubation success before commencing with clinical trials, if appropriate. This is the aim of the Soiled Airway Tracheal Intubation and the Effectiveness of Decontamination (SATIATED) study.

## Author contributions

RP is acting as guarantor for this protocol and conceived the idea for this study. MDT was responsible for development of the statistical analysis planned for SATIATED. MM has provided clinical support and guidance to ensure that this study is feasible to conduct within YAS. RP drafted the initial manuscript, but all authors contributed to the final version.

## Conflict of interest

RP is editor of the *British Paramedic Journal*. MM and MDT declare no conflicts of interest.

## Funding

The study has been funded by a College of Paramedics small research grant. The only condition made by the funder is a request for a copy of the final study report.
